# pH-Dependent Aggregation in Intrinsically Disordered Proteins Is Determined by Charge and Lipophilicity

**DOI:** 10.3390/cells9010145

**Published:** 2020-01-08

**Authors:** Jaime Santos, Valentín Iglesias, Juan Santos-Suárez, Marco Mangiagalli, Stefania Brocca, Irantzu Pallarès, Salvador Ventura

**Affiliations:** 1 Institut de Biotecnologia i Biomedicina and Departament de Bioquímica i Biologia Molecular, Universitat Autònoma de Barcelona, 08193 Barcelona, Spain; jaime.santos@uab.es (J.S.); valentin.iglesias@uab.cat (V.I.); irantzu.pallares@uab.es (I.P.); 2 Galicia Supercomputing Center (CESGA), 15705 Santiago de Compostela, A Coruña, Spain; juansans98@gmail.com; 3 Department of Biotechnology and Biosciences, University of Milano-Bicocca, 20126 Milano, Italy; marco.mangiagalli@unimib.it (M.M.); stefania.brocca@unimib.it (S.B.)

**Keywords:** intrinsically disordered proteins, protein aggregation, protein solubility, amyloids, bioinformatics

## Abstract

Protein aggregation is associated with an increasing number of human disorders and premature aging. Moreover, it is a central concern in the manufacturing of recombinant proteins for biotechnological and therapeutic applications. Nevertheless, the unique architecture of protein aggregates is also exploited by nature for functional purposes, from bacteria to humans. The relevance of this process in health and disease has boosted the interest in understanding and controlling aggregation, with the concomitant development of a myriad of algorithms aimed to predict aggregation propensities. However, most of these programs are blind to the protein environment and, in particular, to the influence of the pH. Here, we developed an empirical equation to model the pH-dependent aggregation of intrinsically disordered proteins (IDPs) based on the assumption that both the global protein charge and lipophilicity depend on the solution pH. Upon its parametrization with a model IDP, this simple phenomenological approach showed unprecedented accuracy in predicting the dependence of the aggregation of both pathogenic and functional amyloidogenic IDPs on the pH. The algorithm might be useful for diverse applications, from large-scale analysis of IDPs aggregation properties to the design of novel reversible nanofibrillar materials.

## 1. Introduction

Protein aggregation is an inherent feature of polypeptides that lies behind the onset of a wide range of human pathologies, including Alzheimer’s and Parkinson’s diseases or type II diabetes [1,2]. Moreover, aggregation often occurs during protein recombinant production and downstream processing, becoming a major bottleneck for the marketing of protein-based drugs [3,4]. Indeed, polypeptides are susceptible to suffering aggregation at every step during protein production, from recombinant expression and purification to formulation and storage [4]. This implies a constant monitorization and optimization of production conditions and processes, which is costly and time-consuming. However, protein aggregation is not always deleterious, and organisms exploit the particular properties of amyloid protein assemblies for beneficial purposes [5,6,7]. This evidence has inspired the use of aggregation-prone proteins and peptides to build up functionalized nanofibrils with applications in tissue engineering, drug delivery or as nanowires and nanosensors [8,9,10,11].

The development of in silico tools able to predict protein aggregation propensities has provided scientists with a versatile toolbox to assist and guide basic research and protein engineering processes [12]. These algorithms exploit the evidence that protein aggregation is driven by short and specific stretches, known as aggregation-prone regions (APRs), displaying unique physicochemical features: low net charge, high hydrophobicity and, frequently, a preference for β-sheet secondary structure [13]. AGGRESCAN, Amylpred, Amyloid Mutants, FoldAmyloid, MetAmyl, PASTA, Tango, Waltz and Zyggregator [14,15,16,17,18,19,20,21,22,23] are some examples of this kind of program. However, most of these prediction methods are blind to the protein environment, despite being well known that factors like temperature, ionic force or pH dramatically impact protein aggregation. Regarding pH, many protein products are purified, stored or formulated at pHs different from 7.0, the default pH in these algorithms. In particular, > 65% of antibodies, Fc fusion products and Fab conjugates are formulated at pH < 6.5 [24,25]. Therefore, it is surprising that, despite the vast experimental data supporting the modulation of intrinsic protein aggregative properties by the solution pH, such an effect has been essentially disregarded in computational approaches [26].

Among the different intrinsic protein properties that can contribute to protein aggregation, hydrophobicity plays a major role. Indeed, APRs usually comprise highly hydrophobic sequence stretches [27,28] and mutations of polar residues to nonpolar ones exacerbate aggregation [29], whereas changes in the opposite direction promote solubility. Therefore, it is not surprising that hydrophobicity is given a major weight, directly or indirectly, in the different equations implemented in sequence-based aggregation predictors [13,30]. Of note, all the aforementioned algorithms assume that the lipophilicity of the sequence is independent of the pH. However, it is well known that the partition coefficients of the neutral and charged species of ionizable amino acids, and therefore their hydrophobicity, depend on the pH of the solution [31,32]. Moreover, the electrostatic properties of proteins, i.e., their net charge in a given solution, are also connected to the solution pH, being important determinants of protein solubility [33,34].

To the best of our knowledge, we present here the first approach to predict how the relative aggregation propensity of a given protein changes with the solution pH. Towards this objective, we exploited a recently developed, pH-dependent lipophilicity scale of amino acids [35] and implemented a simple phenomenological equation that considers the effect of the pH on both the net charge and the lipophilicity of a protein sequence. We assayed the approach on top of intrinsically disordered proteins (IDPs), which lack defined secondary structure elements, to exclude any interference on calculations coming from structural constrains. Our approach accurately predicts the impact of the pH on the aggregation properties of well-known human disease-linked proteins like α-synuclein (α-S), Aβ-40, the islet amyloid polypeptide (IAPP) and the tau K19 variant, as well as in biologically relevant functional amyloids such as the melanosomal protein Pmel17, the B domain of the Bap protein and the corticotropin-releasing hormone, which indicates that it might find general application in the prediction of the pH-dependent aggregation properties of IDPs.

## 2. Materials and Methods

### 2.1. Generation of Lipophilicity Profiles

The pH-dependent lipophilicity scale developed by Zamora and coworkers [35] using continuum solvation calculations was employed to infer the lipophilicity of each individual amino acid at the analyzed pH. Our algorithm employs a sliding window system, as previously described for the AGGRESCAN software, to generate the lipophilicity profile of any given protein [14]. Briefly, the program calculates the average lipophilicity of a sliding window and assigns this value to the amino acid in the center of the window. The size of the window is defined in relation with the protein length: 5 residues for proteins shorter than 75 amino acids, 7 for longer than 75 but shorter than 175, 9 for longer than 175 but shorter than 300 and 11 for longer than 300. The resulting values can be employed to build/draw a lipophilicity profile along the protein sequence or to calculate a mean value of global protein lipophilicity.

### 2.2. Solubility Modelling

The experimental data was obtained from Tedeschi et al. [33]. The pH-dependent experimental solubility of a model IDP was used as training set to parameterize a function that describes protein solubility as a function of pH. We selected two variables to model protein solubility: pH-dependent lipophilicity and net charge. pH-dependent lipophilicity was calculated as the average of the lipophilicity profile. Protein net charge was determined using the protein calculator v3.4 server [36] run at the selected pH. These theoretical values were parameterized against the solubility experimental data using Equation (1):

Solubility = α × Lipophilicity + β × |Net Charge|^2^ + γ × |Net Charge| + δ, (1)

For the parameterization we employed the non-linear least squares approach of the Scipy Python module, being able to define the α, β, γ and δ parameters in Equation (1). Different aggregation studies measure different parameters, from strict solubility to kinetic aggregation constants. To allow comparison between our predictions and the different experimental data, protein solubility is defined here as the inverse of the protein aggregation propensity in a given condition and not as a thermodynamic property of the protein solution.

### 2.3. Data Analysis and Fitting

Kinetic constants for pH-dependent α-S aggregation at 37 °C were obtained from Finke and Morris and Uverski and co-workers [37,38]. Fibrillation rates of IAPP at 25 °C were previously reported by Alexandrescu and colleagues [39]. Tau K19 amyloid formation data at 37 °C was extracted from Jeganathan and co-workers [40]. Data on Aβ40 solubility at 20 °C and different pHs were obtained from Fändrich and co-workers [41]. Data on the effect of pH on functional amyloids were extracted from references [42,43,44]. Linear regression analysis was performed using Graphpad Prism 6. Tendency line and 95% confidence interval were plotted, and regression r-square was added to the graph. For linear regressions, a two-tailed *p*-value was calculated [45].

## 3. Results

### 3.1. Rational Analysis of the Molecular Determinants behind pH-Associated Aggregation

**Figure 1:**
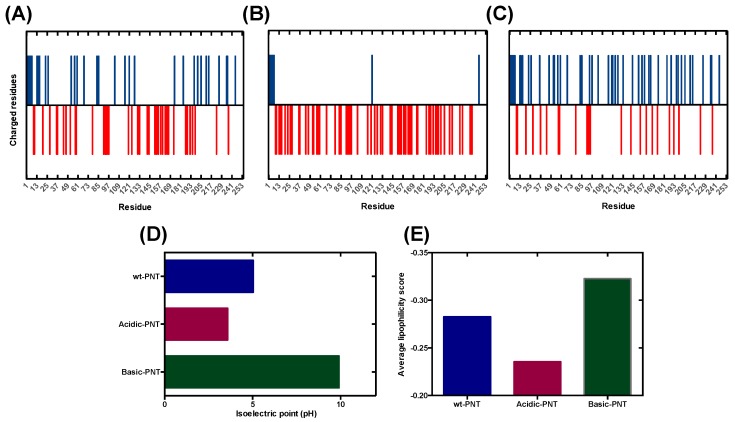
Properties of N-terminus moiety of the measles virus phosphoprotein (PNT) variants. (**A**–**C**) Scheme of charge distribution in wild-type PNT, acidic PNT and basic PNT. Positive and negative residues are represented in blue and red, respectively. (**D**) Isoelectric points of PNT variants. (**E**) Average lipophilicity of PNT variants at pH 7.0.

In order to develop a new theoretical model that can forecast the effect of pH on protein aggregation, we exploited our previous work on the *N*-terminus moiety of the measles virus phosphoprotein (PNT), an IDP model whose aggregation propensity was deeply analyzed in relation to pH and its net charge [33]. We engineered three PNT variants displaying different net charges and isoelectric points (pI) by reversing the sign of charged residues already present in the wild-type sequence, without mutating any other PNT residue (Figure 1A–D and Figure S1). In detail, the acidic PNT has a pI of 3.37 and includes 62 negatively charged residues while basic PNT has a pI of 9.61 and includes 37 positively charged and 23 negatively charged residues. Attempts to produce more basic PNTs, with further unbalanced composition, were unsuccessful. The solubility of each of these protein variants was assessed experimentally across a wide range of pH levels, thus generating an ideal dataset to parametrize a function intended to predict the pH-dependent aggregation propensity of protein sequences [33].

Due to the lack of a well-defined 3D structure, one can hypothesize that the physicochemical determinants of pH-dependent aggregation of IDPs are directly encoded in their amino acid sequence. We propose lipophilicity (hydrophobicity) and net charge as the main properties accounting for the differential aggregation propensity of any given protein at different pH levels. One can argue that this is a rather simplistic approach, but existing methodologies only consider the net charge contribution, while they overlook the role of lipophilicity.

Although unmodified at their apolar residues, our PNT variants exhibit different lipophilicity at neutral pH, since they differ in the identity of the charged amino acids (Figure 1E). In addition, because the hydrophobicity of ionizable amino acids is dependent on the pH, the global protein lipophilicity (average lipophilicity score) in acidic or basic conditions might differ significantly from that calculated at pH 7.0 and this parameter should be taken into account together with the net charge of the polypeptide when forecasting protein solubility.

### 3.2. Analysis and Validation of the Lipophilicity Scale as a Proxy for Aggregation Prediction

**Figure 2:**
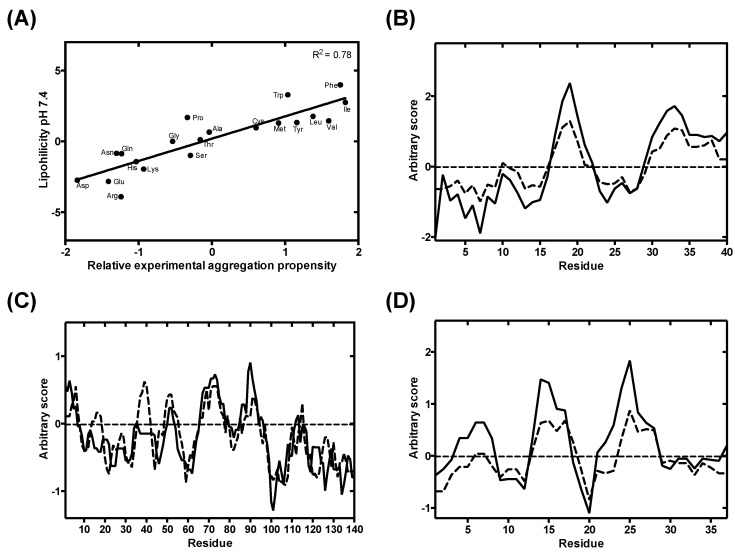
Lipophilicity-based prediction aggregation propensity at pH 7.4 against a state-of-the-art aggregation predictor. (**A**) Linear correlation between amino acids in vivo aggregation propensity, as implemented in AGGRESCAN [15,46], and their lipophilicity at pH 7.4. (**B**–**D**) Overlap between AGGRESCAN-derived (dashed line) aggregation profiles and lipophilicity profiles (solid line) from Aβ40, α-S and IAPP, respectively.

To explore the relationship between amino acid lipophilicity, pH, and protein aggregation, we exploited a pH-dependent amino acid lipophilicity scale recently derived by Zamora and co-workers [35]. We compared the lipophilicity score of each amino acid at physiological pH (pH 7.4) with their in vivo-derived experimental aggregation coefficient [15,46]. We observed a highly significant correlation between aggregation and lipophilicity (*p*-value < 0.00001) (Figure 2A). This is expected, since hydrophobic side chains play a determinant role in aberrant protein self-assembly [47].

Next, we compared the lipophilicity profile of three well-characterized disease-related proteins (Aβ40, α-S and IAPP) at physiological pH with their aggregation profile generated with AGGRESCAN [12]. AGGRESCAN is an in-house developed algorithm, which implements the aforementioned in vivo derived aggregation propensity amino acid scale and stands as one of the most reliable algorithms to predict protein aggregation in close to in vivo conditions [48]. The lipophilicity and aggregation profiles of all the three proteins are in close agreement (Figure 2B–D), indicating that, at constant pH, the lipophilicity can be used as a proxy of aggregation propensity. Although other physicochemical determinants are certainly involved in protein aggregation, we assume here that they have less impact than lipophilicity or charge on pH-modulated protein aggregation.

### 3.3. Modelling pH-Dependent Solubility usIng Lipophilicity and Net Charge

**Figure 3:**
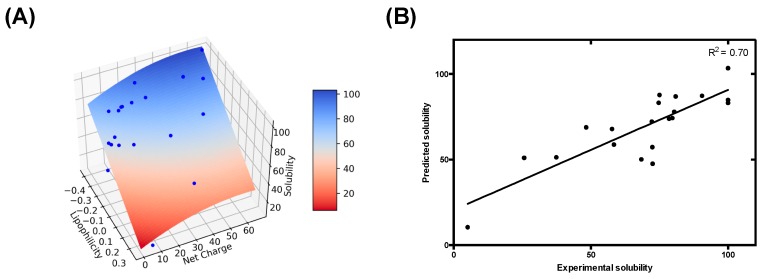
Modeling intrinsically disordered proteins (IDP) pH-dependent solubility based on lipophilicity and net charge. (**A**) Experimental pH-dependent solubility modeled as a 3D surface plot. Experimental data from our previous work is represented as blue dots, and the 3D surface resultant from modeling is colored as a heat map, according to the corresponding predicted solubility as represented in the color bar. (**B**) Correlation between the experimental and predicted solubility. Solid line corresponds to the fit of the data to a linear regression with a *p*-value < 0.00001.

We next sought to build a model to determine the role of lipophilicity and net charge on pH-dependent protein aggregation. For each data point from our previous study with the PNTs, we calculated the protein net charge and the overall protein lipophilicity using a sliding window system analogous to that in AGGRESCAN [14]. Therefore, each data point is defined by its lipophilicity, net charge and experimental solubility, allowing their representation as a 3-axis scatter plot. The visual inspection of their spatial distribution reveals a dispersion that resembles a quadratic 3D surface. Since the distribution shows significant smoothness, we hypothesized that it could be modelled using a function approximated by a Taylor series truncated at second order for the net charge and at first order for the lipophilicity. Thus, we defined an empirical formula (Equation (1)) that describes a bivariate polynomial model with a quadratic component, suitable to address a 3D-surface regression in our dataset. Next, to parameterize this equation, we applied a non-linear least squares approach. As a result of the fitting, we calculated parameters α, β, γ and δ (Table 1). The resulting model, built using Equation (1), delineates a 3D surface, where the solubility is defined as a function of net charge and lipophilicity (Figure 3A). Remarkably, the values derived from the equation show a significant correlation with the observed solubility data (Figure 3B) (*p*-value < 0.00001). In contrast, a mere charge-dependent model, as the one implemented in competing approaches, fail to predict the experimental behavior of the dataset (*p*-value < 0.1) (Figure S2). Overall, these results reinforce the hypothesis that pH-induced lipophilicity fluctuations should be taken into consideration for an accurate prediction of protein aggregation.

### 3.4. pH-Dependent Aggregation Prediction in Disease-Linked Proteins

As a proof of principle of the predictive performance of the approach, we tested our charge and lipophilicity-dependent model in a set of well-characterized IDPs linked with conformational diseases. As discussed, IDPs represent an ideal test set for our model since they allow us to consider almost exclusively the contribution of primary structure on aggregation, excluding the folding and protein stability contributions. The obtained pH-dependent aggregation profile for each protein was compared with available experimental data in the literature by assessing the linear regression between experimental and predicted solubility values.

#### 3.4.1. α-Synuclein (α-S)

**Figure 4:**
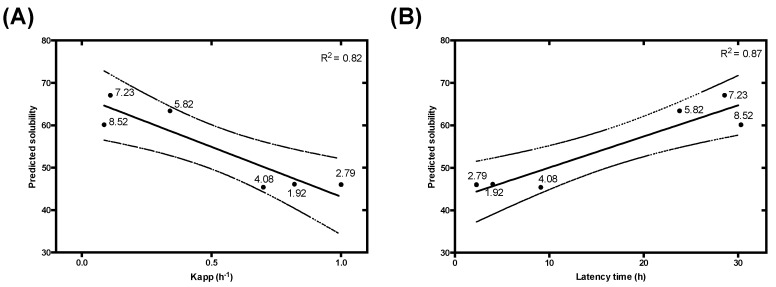
Prediction of experimental α-synuclein (α-S) aggregation kinetic constants. Correlation between the elongation constant Kapp (**A**) and latency time (**B**) and the predicted protein solubility at different pH (1.92, 2.79, 4.08, 5.82, 7.23, 8.52). Experimental data were extracted from Morris and Finke’s work [37]. Each point represents an experimental datum labelled with its corresponding pH. A linear regression (solid line) and its 95% confidence interval (dashed line) were applied to fit the data with a *p*-value < 0.05 for Kapp and < 0.01 for latency time.

Parkinson’s disease (PD) is the second most prevalent neurodegenerative disorder. Brains from PD patients exhibit the recurrent presence of intracellular proteinaceous aggregates, mainly composed by α-S. These deposits, known as Lewy bodies, represent the main neuropathological hallmark of the disease and are responsible for eliciting cellular toxicity and causing neuronal death [49,50,51]. From a molecular perspective, α-S is a 140-residues IDP, highly expressed in the synapses of dopaminergic neurons that has been shown to assemble in vitro into amyloid fibrils under different conditions [52,53,54]. Owing to the connection between α-S and PD, there is a great interest to explore the determinants of α-S aggregation. In that context, Uversky and co-workers described the effect of pH on α-S solubility [38]; later on, Finke and Morris fitted the data into formal aggregation kinetic equations [37]. To assess whether the effect of pH on α-S aggregation could be anticipated by our equation, we compared the predicted α-S solubility with the experimental aggregation kinetic data parameters across a wide range of pHs. We found an excellent correlation between our predicted solubility and both the elongation constants and latency times of the reaction (Figure 4). Remarkably, in α-S the majority of the charged residues are segregated in the C-terminal of the protein, while the hydrophobicity is clustered in its central NAC domain; such dual distribution does not seem to compromise the performance of the approach.

#### 3.4.2. Islet Amyloid Polypeptide (IAPP)

**Figure 5:**
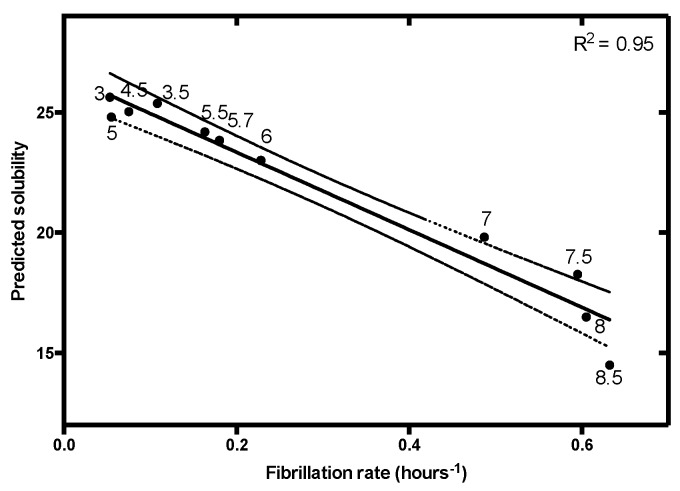
Linear correlation between islet amyloid polypeptide (IAPP) fibrillation rate and predicted solubility at different pH (3, 3.5, 4.5, 5, 5.5, 5.7, 6, 7, 7.5, 8.5, 9). Data on IAPP fibrillation were extracted from Alexandrescu and co-workers [39]. Data were fitted to linear regression (solid line) with a *p*-value < 0.00001 and its 95% confidence interval was represented (dashed line).

Aggregates of IAPP are present in the extracellular space of the islet of Langerhans in patients suffering from type II diabetes [55]. IAPP is an intrinsically disordered peptide hormone co-stored with insulin and involved in glycemic control [56]. Under pathological conditions, IAPP forms extracellular amyloid deposits causing the degeneration of pancreatic β-cells [57]. This behavior is thought to be dependent on the environmental pH [58,59], with the low pH of the secretory granules (pH ≈ 5.5) able to protect IAPP from aggregation, and the extracellular environment pH (pH ≈ 7.4) pro-aggregational. The pH-dependent fibrillation of IAPP was studied by Alexandrescu and co-workers, uncovering a strong pH dependency for this peptide [39]. This work provided us with a complete set of kinetic data over a wide pH range to further test our model. IAPP fibrillation rates are tightly connected to the solution pH, a trend that can be predicted with high confidence by applying our equation (Figure 5).

#### 3.4.3. Alzheimer’s Disease Related Proteins: Amyloid-Beta Peptides and Tau Protein

**Figure 6:**
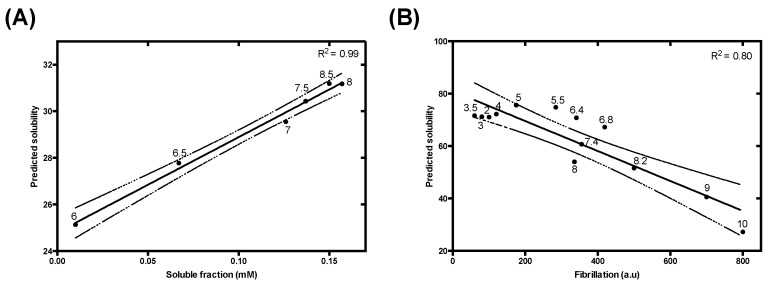
Analysis of the effect of pH variations on (**A**) Aβ-40 and (**B**) tau K19 variant solubility. (**A**) Correlation between Aβ-40 predicted and experimental solubility at different pH (6, 6.5, 7, 7.5, 8, 8.5). Experimental data were extracted from Fändrich and coworkers [41]. (**B**) Analysis of the experimental amyloid formation reported by Thioflavin S fluorescence emission, extracted from Jeganathan and co-workers at a range of pH from 3 to 10 [40]. Data were fitted to linear regression (solid line) and its 95% confidence interval was represented (dashed line), with a *p*-value < 0.0001 in both cases.

Alzheimer’s disease (AD) is the most prevalent neurodegenerative disorder and is characterized by a progressive cognitive impairment. The molecular pathology of AD is characterized by the combined presence of two aberrant protein deposits in brain tissue: extracellular amyloid deposits (amyloid plaques) and intraneuronal neurofibrillary tangles [60]. The β-amyloid peptides Aβ-40 and Aβ-42 are intrinsically disordered proteolytic fragments of amyloid-beta precursor protein [61] and their aggregates constitute the principal components of the amyloid plaques. Tau is an IDP [62,63] whose main function is promoting microtubule assembly and stability. In AD, tau aggregation results in the assembly of abnormal neurofibrillary tangles. The aggregation reactions of these proteins have been extensively characterized due to their pivotal role in AD. Fändrich and co-workers addressed the effect of pH over Aβ-40 solubility, reporting a significant decrease in solubility below neutral pH [41]. Our model is able to recapitulate this pH-dependence of Aβ-40 solubility with high accuracy (Figure 6A). Jeganathan and co-workers studied how pH affected tau K19 aggregation [40]. Tau K19 is a truncated construct containing three microtubule binding repeats (R1, R3, and R4), whose aggregates show structural features that are reminiscent of those of the full-length tau protein [64,65]. Again, our algorithm successfully models the experimental behavior of tau K19 aggregation at different pH levels (Figure 6B).

#### 3.4.4. Use of a Lipophilicity Term Improves Accuracy in the Prediction of the pH-Dependent Aggregation of Disease-Linked Proteins

The protonation and deprotonation of ionizable amino acids causes changes in both lipophilicity and net charge. Therefore, it is expected that both parameters are somehow correlated, since both properties depend on the pKa of the amino acid. We assessed if the continuous and fractional description of the dependence of the net charge on the pH we use here can predict the pH-dependent aggregation of disease-linked proteins in the absence of a lipophilicity term (Table 2).

The net charge is a reasonable predictor of pH-dependent aggregation in two conditions: (i) When along the experimental range of pH, the protein remains positively charged (below its pI), as for IAPP (all pHs) and K19 (13 of 14 analyzed pHs). In this case, the net charge decreases with increasing pH, whereas the lipophilicity has the opposite trend and both factors contribute to an increased aggregation at higher pHs. (ii) When the protein remains negatively charged in the analyzed pH range (above its pI), as for Aβ-40 (all pHs). In this case, both the net charge and the lipophobicity increase with increasing pH, having an opposite impact on protein aggregation.

The net charge is a poor predictor of pH-dependent aggregation when the range of experimentally analyzed pH levels comprise values that are both below and above the protein pI, like in the case of α-S or the above-discussed PNTs. In this case, lipophilicity increases continuously with the pH, whereas the net charge decreases as the pH approaches the pI and increases when it moves away from it. Thus, at values below the pI, net charge and lipophilicity display opposite trends whereas above the pI they exhibit the same tendency.

Importantly, our equation remains predictive in the three above-mentioned regimes and performs equal or better than the net charge term alone for any considered protein (Table 2).

### 3.5. Predicting the Impact of pH on the Aggregation of Functional Amyloids: Context-Dependent Aggregation to Confine Functional Self-Assembly

Amyloid fibrils have been traditionally considered pathogenic agents responsible for a set of devastating human disorders, such as the aforementioned examples. However, during the last decade, a large body of evidence supports the idea that the amyloid architecture can be exploited to develop biological functions [66]. Functional amyloids work under physiologically conditions without any associated cytotoxicity [67,68], mainly because, in contrast to their toxic counterparts, coordinated cellular strategies have been evolved to control their assembly. One of these strategies consists in confining aggregation inside a specific cellular compartment in a pH-dependent manner. This natural strategy provides an exceptional benchmark to validate our predictive model.

#### 3.5.1. Pigment Cell-Specific Melanosome Protein

**Figure 7:**
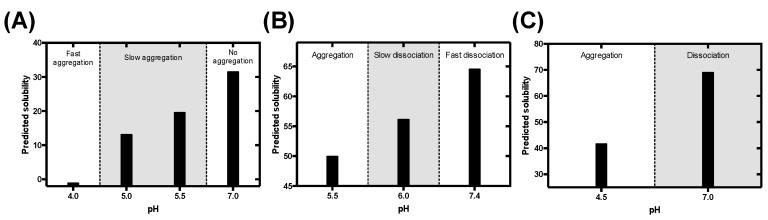
Evaluation of the pH-dependent mechanism of fibrillation of functional amyloids. Pigment cell-specific melanosome protein (Pmel17) (**A**), corticotropin-releasing hormone (CRF) (**B**), and BapB (**C**) predicted solubility compared with their physiological fibrillation and disaggregation tendencies. The different regions of aggregation are delimited by dotted lines.

The pigment cell-specific melanosome protein (Pmel17) is involved in the biogenesis and maturation of melanosomes, organelles specialized in melanin synthesis, present in melanocytes and epithelial cells in mammals. The specific role of Pmel17 is the formation of amyloid fibrils in the lumen of the melanosomes that optimize the sequestration and condensation of melanin [39,65]. Pmel17 fibrillation occurs in the acidic environment of the early stage melanosome (pH ≈ 4–5). Lee and co-workers first reported the amyloidogenesis of the repeat domain (RPT) of Pmel17, describing a strong dependence on solution pH: a fast aggregation at pH 4, slower at pH 5 and 5.5 and no aggregation, and even fibril disaggregation, at pH 7 [42]. Our algorithm successfully discriminates those three regimens of aggregation (Figure 7A).

#### 3.5.2. Corticotropin-Releasing Hormone

Maji and coworkers discovered in 2009 a novel activity of functional amyloids as storage of peptide hormones in secretory granules [43]. They described that peptide hormones fibrillate due to the low pH (≈ 5.5) of those granules and that, upon release to the extracellular environment (pH ≈ 7.4), the fibrils gradually disassemble into the monomeric bioactive specie. In that work, they explore this effect in vitro on the corticotropin-releasing hormone (CRF) by inducing the formation of fibrils at pH 5.5, and analyzing their disaggregation at different higher pHs (pH 6 and 7.4). The experimental disaggregation is faster at pH 7.4. This behavior, with a gradual gain of solubility at increasing pHs and fast dissociation at pH 7.4, is fairly recapitulated by our model (Figure 7B).

#### 3.5.3. B Domain of the Bap Protein

*Staphilococcus aureus* Bap is an extracellular protein able to self-assemble at acidic pH (≈ 4.5), forming amyloid fibrils that scaffold the formation of a biofilm matrix [44]. In the case of Bap, aggregation is confined to the extracellular environment where it acts as a pH sensor and, upon acidic conditions, orchestrates a multicellular response that elicits biofilm formation. Lasa, Valle and co-workers reported the aggregation of this protein, identified an amyloidogenic domain (BapB) and characterized its pH-dependent aggregation [44]. BapB forms amyloid fibrils at pH 4.5 that dissociate when the pH rises to attain the neutrality. Once more, our approach is able to predict such behavior (Figure 7C).

## 4. Discussion

In the last decades, the advances in the field of protein aggregation have resulted in the development of over 40 different predictive methods to computationally assess protein deposition. Thus, we have at our disposal a wide variety of algorithms based on conceptually different molecular determinants to systematically predict protein aggregation. However, these approaches barely exploit the influence of the protein environment. This is important because solvent conditions impact solubility by modulating the hydrophobic effect, electrostatic interactions or the degree of protonation of the different ionizable groups. Here, we presented a novel phenomenological model whose aim is the evaluation of protein solubility as a function of solvent pH. Exploiting our previous experimental data on the solubility of a charge-engineered model IDP, we were able to weigh the contribution of lipophilicity and net charge to protein solubility and, subsequently, elaborate a phenomenological predictor with high accuracy in predicting pH-dependent aggregation of IDPs. Our results indicate that in addition to the net charge, pH also modulates protein lipophilicity and that such control has a significant impact on protein solubility.

Our algorithm demonstrates high accuracy in predicting pH modulation of aggregation propensity in a set of disease-associated IDPs, such as α-S, IAPP, tau K19 fragment and Aβ-40. Moreover, we employed our approach to evaluate the aggregation propensity of three proteins reported to form functional amyloids in vivo upon pH shifts. Interestingly enough, in these proteins, evolution has exerted a selective pressure to attain a reversible fibrillation mechanism where pH controls the assembly and disassembly of the fibrils. We were able to predict such behavior by analyzing only protein primary structures, highlighting that this conformational transition is intrinsically imprinted in the polypeptide chain.

The main application of our prediction method would be the profiling of protein solubility along a continuous pH interval, since it demonstrates a remarkable accuracy in describing this behavior. Indeed, the approach delineates a sequence profile at any desired pH, allowing us to assess the protein regions that contribute the most to the pH-dependent aggregation of a given protein. Electrostatic and hydrophobic interactions are variably influenced by temperature and thus, we cannot argue that the model will be predictive at any pH/temperature combination. However, this temperature dependence can be likely included in the equation if the solubility of our designed IDPs at different temperatures is experimentally measured.

The model is simple, and computation is fast, which should allow the analysis of large sequence datasets, including the complete complement of IDPs in a given proteome. It would be interesting to assess whether the IDPs residing in cellular compartments are optimized to display the maximum solubility at the specific compartment pH. The algorithm can also contribute to understanding the role of changes in intracellular pH in protein phase separation reactions, since this phenomenon results from the coalescence of intrinsically disordered protein regions [69]. We also propose that our method may have an impact in the design of nanomaterials with pH-modulable assembling properties, which can transition between soluble and amyloid-like states simply by shifting the solution pH.

The method can also be used to assist the purification, formulation and storage of proteins of biotechnological and therapeutic interest, by predicting the range of pH in which they are more soluble, as long as they are intrinsically disordered, as in the case of peptidic hormones.

It is important to note that, in its present formulation, the method is not intended for the prediction of the pH-dependent aggregation of globular proteins, like therapeutic antibodies or the prion protein, from their initially folded states. The concept should be first implemented in a structural predictor, where the intrinsic charge and lipophilic properties of amino acids would be modulated according to the protein conformational properties at any given pH. This step will be analogous to the evolution of AGGRESCAN [14] into our structural A3D aggregation predictor [70,71,72] and thus, perfectly attainable.

## Figures and Tables

**Table 1:** Fitting parameters resulting from the non-linear least squares parametrization.

Parameter	α	β	γ	δ
**Values**	−97.82	−0.00747	0.8770	38.24

**Table 2:** Comparison between correlation values (R^2^ and *p*-value) obtained with a charge or a charge and lipophilicity dependent model. Best parameters for a given protein in bold, values not significant at *p* < 0.05 in italics. * The value inside brackets corresponds to the protein pI.

Protein		PNTs	α-S (4.67) *		IAPP	Aβ40	Tau K19
			Kapp	Tlag	(8.90) *	(5.31) *	(9.68) *
**Charge**	R^2^	0.20	0.47	0.50	0.86	0.93	**0.80**
	*p*-value	0.048	*0.13*	*0.12*	0.000041	0.0019	**0.000037**
**Charge and Lipophilicity**	R^2^	**0.70**	**0.82**	**0.87**	**0.95**	**0.99**	**0.80**
	*p*-value	**<0.00001**	**0.013**	**0.0066**	**<0.00001**	**0.000039**	**0.000037**

## Supplementary Materials

The supplementary materials are available online at https://www.mdpi.com/2073-4409/9/1/145/s1.
