# Supplementary material for: pH-Dependent Aggregation in Intrinsically Disordered Proteins Is Determined by Charge and Lipophilicity
Source: Cells. 2020 Jan 8;9(1):145. doi: 10.3390/cells9010145 (PMC7017033; doi:10.3390/cells9010145)

Supplementary material

S1: Sequence of the PNTs variants

We used the N-terminus moiety of measles virus phosphoprotein (PNT) as a model IDP. Acidic and basic variants were obtained as described in [1]. Briefly, basic (H, K, R) residues from the wild type protein were substituted with acidic (E or D) in the acidic variant; while in the basic variant wild type acidic residues were almost all substituted by basic ones.

```
PNT wild type (N-terminus moiety of measles virus phosphoprotein)
MHHHHHHAEEQARHVKNGLECIRALKAEPIGSLAIEEAMAAWSEISDNPGQERATCREEK
AGSSGLSKPCLSAIGSTEGGAPRIRGQGPGESDDDAETLGIPPRNLQASSTGLQCHYVYD
HSGEAVKGIQDADSIMVQSGLDGDSTLSGGDNESENSDVDIGEPDTEGYAITDRGSAPIS
MGFRASDVETAEGGEIHELLRLQSRGNNFPKLGKTLNVPPPPDPGRASTSGTPIKKENLY
FQGSHMPGTMPGTM

PNT acidic variant
MHHHHHHAEEQADDVENGLECIEALDAEPIGSLAIEEAMAAWSEISDNPGQEDATCEEEE
AGSSGLSEPCLSAIGSTEGGAPDIDGQGPGESDDDAETLGIPPENLQASSTGLQCDYVYD
HSGEAVDGIQDADSIMVQSGLDGDSTLSGGDNESENSDVDIGEPDTEGYAITDEGSAPIS
MGFDASDVETAEGGEIEELLELQSDGNNFPELGDTLNVPPPPDPGEASTSGTPIDDENLY
FQGSHMPGTMPGTM

PNT basic variant
MHHHHHHAEEQARHVKNGLECIRALKAEPIGSLAIKEAMAAWSEISRNPGQKRATCREEK
AGSSGLSKPCLSAIGSTEGGAPRIRGQGPGESDRDAKTLGIPPRNLQASSTGLQCHYVYR
HSGKAVKGIQDARSIMVQSGLDGRSTLSGGRNESRNSRVDIGKPRTEGYAITDRGSAPIS
MGFRASDVKTAEGGKIHELLRLQSRGNNFPKLGKTLNVPPPPDPGRASTSGTPIKKENLY
FQGSHPGTMPGTM
```

References

1. Tedeschi, G., Mangiagalli, M., Chmielewska, S., Lotti, M., Natalello, A. & Brocca, S. (2017) Aggregation properties of a disordered protein are tunable by pH and depend on its net charge per residue, Biochimica et biophysica acta General subjects. 1861, 2543-2550.

Figure S2: Correlation between charge distribution and change in solubility in a range of pH for PNTs

Correlation between the experimental solubility and net charge variation. Solid line corresponds to the fit of the data to a linear regression with a p-value < 0.05.

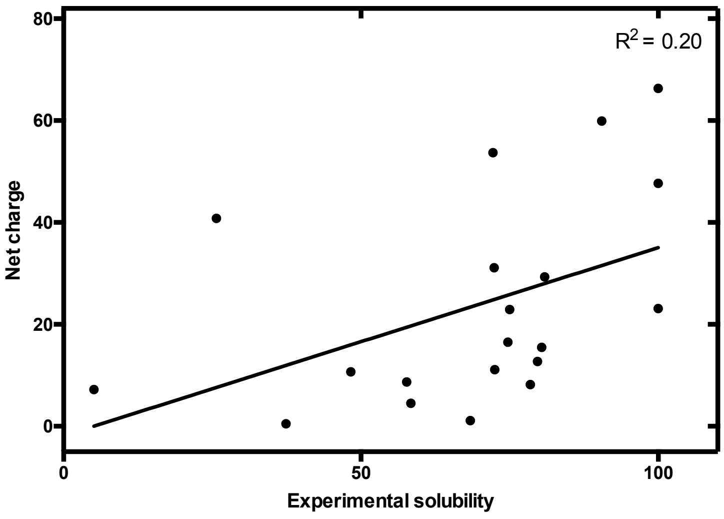

Supplement: Supplementary file 1 [file cells-09-00145-s001.pdf]
